# What Led to the Nigerian Boycott of the Polio Vaccination Campaign?

**DOI:** 10.1371/journal.pmed.0040073

**Published:** 2007-03-20

**Authors:** Ayodele Samuel Jegede

## Abstract

Jegede discusses the recent controversy surrounding polio immunization in Nigeria, in which three northern states boycotted the immunization campaign.

Vaccination is a crucial tool for preventing and controlling disease, but its use has been plagued by controversies worldwide [1–6]. In this article, I look at the controversy surrounding the immunization program against polio in Nigeria, in which three states in northern Nigeria in 2003 boycotted the polio immunization campaign. I discuss the problems caused by the boycott, its implications, and how it was resolved. Finally, I make recommendations for the future to prevent a similar situation from arising.

## Methods

For this article, I consulted relevant books, journals (online and print), Internet materials, and newspaper articles. In particular, I also searched for documentary materials on the history of vaccination in northern Nigeria, factors responsible for the boycott, and ethical issues arising from the boycott.

## The Kick Polio Out of Africa Campaign

Due to the difficulty faced by some national governments in containing polio outbreaks, the World Health Organization (WHO) in 1988 launched the Global Polio Eradication Initiative (GPEI) with the goal of eradicating the disease by the year 2000 (see [7] and http://www.polioeradication.org). In 1989, the World Health Assembly approved a global plan of action for eradicating polio and the WHO Regional Committee for Africa adopted the resolution [8].

**Figure 1:**
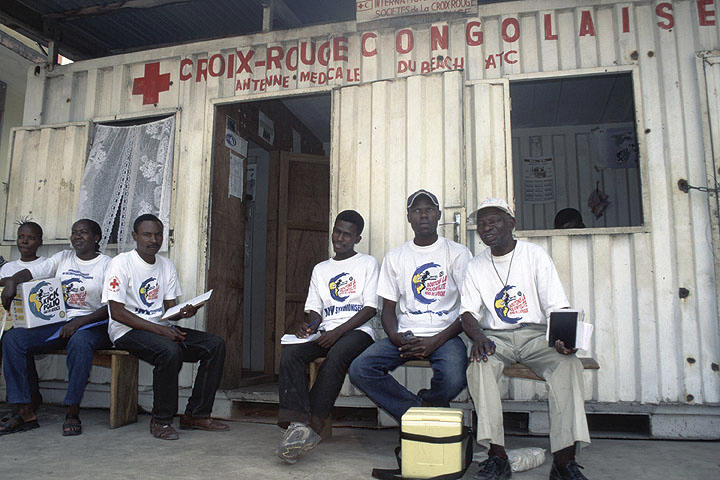
The Kick Polio Out of Africa Campaign This photo shows Red Cross volunteers, wearing “Kick Polio Out of Africa” T-shirts, waiting for boat transportation from Kinshasa, the Democratic Republic of the Congo, on a National Immunization Day. (Photo: Global Polio Eradication Initiative)

**Figure 2:**
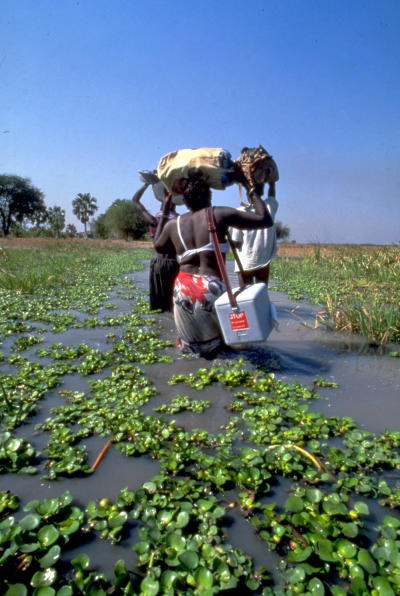
A Vaccination Team Crosses a River to Reach a Village During a National Immunization Day (Photo: Global Polio Eradication Initiative)

The WHO Regional Committee for Africa intensified its polio eradication strategies in 1996. Nelson Mandela launched the “Kick Polio Out of Africa” campaign (Figure 1) [9], which aimed to vaccinate 50 million children in 1996 alone. Mass immunization campaigns were boosted by National Immunization Days (Figure 2), acute flaccid paralysis surveillance, training of community health workers at the local level, and door-to-door campaigns [10,11].

The Kick Polio Out of Africa campaign assured functional cold chain systems and continuing education of communities about the importance of routine immunization [9]. From 1997, through an alliance with the African Football Confederation, leading African football players have participated in public awareness campaigns by distributing posters, conducting radio interviews, and holding public autograph sessions [8].

In mid-October 2003, the GPEI launched what was hoped to be the final onslaught against polio, with a plan to immunize more than 15 million children in west and central Africa. The GPEI had particular concerns about the high prevalence of polio in Nigeria, which accounted for 45% of polio cases worldwide and 80% of cases reported from the African region in 2003 [12]. This high prevalence was attributed to poor vaccine coverage during the previous control campaigns. But the GPEI's hopes were dashed by a boycott of the polio immunization campaign in three states in northern Nigeria, amidst rumours and public distrust.

## The Boycott in Northern Nigeria

Public trust is essential in promoting public health [13]. Such trust plays an important role in the public's compliance with public health interventions, especially compliance with vaccination programs, which target mainly healthy people. Where public trust is eroded, rumours can spread and this can lead to rejection of health interventions.

In northern Nigeria in 2003, the political and religious leaders of Kano, Zamfara, and Kaduna states brought the immunization campaign to a halt by calling on parents not to allow their children to be immunized. These leaders argued that the vaccine could be contaminated with anti-fertility agents (estradiol hormone), HIV, and cancerous agents.

In an article reported by News24.com, a South African online news Web site, Sule Ya'u Sule, speaking for the governor of Kano, is quoted as saying: “Since September 11, the Muslim world is beginning to be suspicious of any move from the Western world…Our people have become really concerned about polio vaccine” [14]. In the same article, Datti Ahmed, a Kano-based physician who heads a prominent Muslim group, the Supreme Council for Sharia in Nigeria (SCSN), is quoted as saying that polio vaccines were “corrupted and tainted by evildoers from America and their Western allies.” Ahmed went on to say: “We believe that modern-day Hitlers have deliberately adulterated the oral polio vaccines with anti-fertility drugs and…viruses which are known to cause HIV and AIDS” [14].

The *New York Sun* reported that this fear of polio vaccines in northern Nigeria “caught on because of the war in Iraq” [15]. Ali Guda Takai, a WHO doctor investigating polio cases in Kano, told the *Baltimore Sun*, “What is happening in the Middle East has aggravated the situation. If America is fighting people in the Middle East, the conclusion is that they are fighting Muslims” [16].

Embarrassed by the political undertone of the boycott, the prominent Islamic scholar Sheikh Yusuf Al-Qaradawi, President of the International Fiqh Council, said: “In fact, I was completely astonished about the attitude of our fellow scholars of Kano towards polio vaccine. I disapprove of their opinion, for the lawfulness of such vaccine in the point of view of Islam is as clear as sunlight” [17]. Sheikh Qaradawi said that the same polio vaccine has been effective in over 50 Muslim countries, and blamed the SCSN for creating a negative image of Islam: “They distort the image of Islam and make it appear as if it contradicts science and medical progress” [17].

## Background to the Boycott

### The historical context

The polio vaccination boycott should not be considered in isolation, but rather in the context of the history of orthodox health services in northern Nigeria. Generally, utilization rates of orthodox health-care services in the region have always been low. For instance, comparative utilization rates of southern Nigeria versus northern Nigeria were 50% versus 18% in 1990 (i.e., half of people in the south used orthodox health services, compared with less than one fifth in the north), 60% versus 11% in 1999, and 64% versus 8% in 2003 [18–20].

Other historical factors that fed into the polio vaccination boycott include population and fertility regulation. In the 1980s, President Babangida's administration adopted a population policy that set a limit of four children per woman. Some people connected this population control campaign with immunization, believing that vaccination was one way the government might be reducing the population [21]. This belief was not restricted to northern Nigeria—similar opinions were also expressed in some communities in southern Nigeria.

For example, in an anthropological study carried out in Nigeria [22], an adult male participant stated that “people do carry rumour that immunisation is a secret way of controlling population.” A young female participant said “some people say that immunisation is part of the methods used to check the number of children a woman can bear.”

Another important factor that played a role in the polio vaccine boycott was the general distrust of aggressive, mass immunization programs in a country where access to basic health care is not easily available [16]. In his report for the *Baltimore Sun*, John Murphy wrote: “The aggressive door-to-door mass immunizations that have slashed polio infections around the world also raise suspicions. From a Nigerian's perspective, to be offered free medicine is about as unusual as a stranger's going door to door in America and handing over $100 bills. It does not make any sense in a country where people struggle to obtain the most basic medicines and treatment at local clinics” [16].

### The political context

In Nigeria, states have administrative control over health affairs at the primary and secondary care levels while the federal government has control at the tertiary care level. Although the federal government sets health policy for the nation, immunization is under the primary health-care system controlled by each state government. This was why the Kano state government was able to issue a directive to halt the immunization exercise planned by the federal government.

Nigeria being a multiparty society, opposition parties exercise their political rights by constantly challenging the ruling party. After the May 2003 presidential elections the All Nigeria People's Party (ANPP) led by General Muhammadu Buhari, one-time military dictator and Head of State, filed a case in Nigeria's Supreme Court challenging the victory of President Olusegun Obasanjo of the People's Democratic Party (PDP) [23]. Also, Kano as a state under the control of the ANPP challenged the polio vaccination exercise organized by the PDP-controlled federal government.

Nigeria is undergoing a political transition from a northern-led military regime to a southern-led democracy. Until 1999, the north had ruled the country for more than 30 of the 46 years of independence. Since the beginning of the new democratic system of government in 1999, power shifted to the south (specifically the south-west). These changes have resulted in political tensions between the south and north. These tensions might explain why the religious leaders in northern states who boycotted the polio immunization campaign believed that the southern-led federal government was acting in the interests of Western powers. The northern and southern parts of the country had different colonial experiences. While the north was colonized by the Islamic Jihadists, the south was colonized by the British. These colonial experiences are responsible for political differences between north and south and different attitudes to modern medicine.

### The Trovan trial

Suspicions about Western health interventions were already circulating in northern Nigeria, ahead of the polio vaccination boycott, in the wake of Pfizer's 1996 “Trovan trial” [24–26]. The trial was discussed in detail in a *BMJ* feature entitled “Pfizer accused of testing new drug without ethical approval” [24].

In brief, the *BMJ* reports that in 1996 Pfizer sent a team to Kano during an epidemic of meningococcal meningitis. To test the efficacy of its new antibiotic trovafloxacin (Trovan), the team conducted an open-label trial in 200 children—half were given the gold standard treatment for meningitis, ceftriaxone, and half received trovafloxacin. Five of the children given trovafloxacin died, together with six who were given ceftriaxone. The *BMJ* reported: “*The Washington Post* has been investigating the trial and alleges that at least one child was not taken off the experimental drug and given the standard drug when it was clear that her condition was not improving—which is against ethical guidelines.” The *BMJ* reported that the Nigerian health minister appointed a federal investigative panel to determine whether the trial was conducted legally, and if so, whether it was morally right.

On May 7, 2006, *The Washington Post* reported that it had been privileged to see a secret report of the panel's investigation, which alleged that Pfizer undertook an “illegal trial of an unregistered drug” when the company enrolled children into the Trovan trial [27]. In response to the leaked report, Pfizer issued a press statement saying: “Pfizer is confident that no one associated with the Trovan clinical study—conducted in Kano, Nigeria during a meningitis epidemic in 1996—ever put a patient's health at risk and that the company acted in the best interests of the children involved in the study, using the best medical knowledge available” [28].

In 2001, 30 Nigerian families sued Pfizer in a federal court in New York [29]. The suit alleged: “Pfizer chose to select children to participate in a medical experiment of a new, untested and unproven drug without first obtaining their informed consent.” During the following four years, Pfi zer argued that the case should not be heard in a United States court at all [30]. In August 2005, Southern District of New York Judge William H. Pauley III agreed, ruling that Nigeria, not the US, was the proper place to try a lawsuit over Pfizer's conduct in the Trovan trial [31].

According to John Murphy's report for the *Baltimore Sun*, the Trovan trial may have left some Nigerians distrustful of Western interventions: “Some of Kano's fears of the vaccine stem from its experience with the U.S. pharmaceutical giant Pfizer Inc.” [16].

## Federal Response to the Polio Boycott

In response to public outcry about the polio vaccine, the Nigerian federal government set up a technical committee on October 29, 2003 to assess the safety of the polio vaccine, sending samples of the vaccine for laboratory tests abroad. The committee's report, however, was rejected by the SCSN, which alleged that the Muslim community was not properly represented on the committee.

In response to this allegation, the federal government appointed another technical committee, which included selected members of the Muslim group Jama'atu Nasril Islam, to further reconfirm the safety of the vaccine. But the SCSN again rejected the nominees, asking for the inclusion of its own nominees. Justifying its continued opposition to the polio vaccine despite the alarming 30% increase in polio prevalence, in January 2004, the Kano State Government argued: “…a lesser of two evils, to sacrifice two, three, four, five even ten children to polio than allow hundreds of thousands or possibly millions of girl-children likely to be rendered infertile” [17].

Although the truth of the rumour that the polio vaccine contained HIV and cancerous and anti-fertility agents was never established, the lack of trust among the general population in northern Nigeria about the efficacy of Western medicine remained. All efforts by the federal government to dispel the rumours were rejected. For instance, in April 2004 Datti Ahmed argued that “the SCSN harbours strong reservations on the safety of our population, not least because of our recent experience in the Pfizer scandal, when our people were used as guinea pigs with the approval of the Federal Ministry of Health, and the relevant UN agencies” [17].

This impasse was eventually resolved in July 2004 through dialogue, with religious leaders playing a significant role in the process. The federal government had invited political and religious leaders to a series of meetings in order to find a solution to the impasse. The WHO and UNICEF also played a role in breaking the deadlock. These meetings led to a consensus in February 2004 to accept the SCSN's demand to test the vaccine independently in a Muslim country. In February 2004, the Nigerian government sent state and religious representatives to South Africa, Indonesia, and India to observe testing of the polio vaccine and “bring back proof” that it was not contaminated with HIV [17].

In defence of the 11-month boycott, the Kano state governor, Ibrahim Shekarau, reaffirmed that their decision was influenced by unsatisfactory test results by the federal government's teams. Satisfied by the quality and process of production of the polio vaccine, the Kano state team returned with a seal of approval from Biopharma, an Indonesian company, which they later recommended be the new supplier of polio vaccines for the predominantly Muslim states and perhaps the rest of the country [17]. Indonesia is a Muslim country trusted by Nigeria's Muslim leaders for testing the polio vaccine.

Two months after Kano state resumed its polio immunization program, about 150 Muslim clerics and traditional chiefs from Chad, Cameroon, Niger, Togo, Benin, and Burkina Faso met in Kano on September 22, 2004 to discuss the way forward in respect to the polio immunization campaign. The meeting was hosted by WHO and UNICEF, and its aim was to “inform religious and traditional leaders about issues that affect children, with emphasis on polio.” The meeting also shared knowledge and experiences and generated an advocacy agenda to ensure that the right messages were delivered to the people [32,33].

## African Communitarianism

The federal government, having lost the public trust of one state—a loss of trust fuelled by Muslim leaders—became handicapped in providing health services. In many parts of Africa, communication and authority flow downward from community leaders who are the gate keepers and decision makers. In the Hausa community of northern Nigeria, traditional rulers have powers derived from both culture and religion. This gives them the opportunity to perform both political and religious roles. They rule through the traditional and the Islamic councils, under the supervision of the local government, having their received staff of office from the state government.

The modern political system in northern Nigeria relies on this structure to reach out and mobilize people. This is important in the sense that it shows a type of communitarianism where individual autonomy cannot be separated from that of the community [34]. The Kano state leaders believed that they were acting to protect the interest of their people, though unfortunately, they ended up disrupting national and global health agendas.

## The Future of Immunization Efforts

A fresh outbreak of polio was reported in Kano in October 2003. The BBC reported that, due to this fresh outbreak, a new strain of the polio virus was traced to other parts of the country [35]. Even many years after the boycott, polio outbreaks remain a regular occurrence in Nigeria, and these show some form of resistance to vaccines. While three or four doses of polio vaccine administered to a young infant are enough to provide protection in most parts of the world, in Nigeria, with so much polio virus circulating, children under five years must be immunized up to eight or more times [23].

The recent case of an 18-month-old boy in Nasarawa in Kano shows that there is ongoing suspicion about the polio vaccine in Nigeria. The boy was considered likely to have polio, having lost the use of one of his legs after being diagnosed with acute flaccid paralysis. His mother confirmed that he had never been vaccinated because her husband did not allow it [23]. It is still a major undertaking to have the population understand that the vaccine is safe [36].

The September 2006 report of the WHO Regional Committee for Africa indicated that “there continues to be very high-intensity wild poliovirus transmission in the remaining endemic country in the region. The number of confirmed polio cases in northern Nigeria more than doubled in the first five months of 2006 as compared to the same period in 2005. Nigeria now accounts for over 80% of the 2006 global polio burden” [37,38].

New cases of polio have recently been reported in several west and central African countries that were previously free of polio: Benin, Burkina Faso, Cameroon, Central African Republic, Chad, Côte d'Ivoire, Ghana, and Togo. *The Lancet* recently reported that “poliovirus serotype 1 caused a very serious and large scale outbreak during 2004 in western and central Africa (spreading from Nigeria) where vaccination was refused for political or theological reasons, or fear of deliberate contamination of the vaccine with HIV or infertility agents” [38].

**Figure 3:**
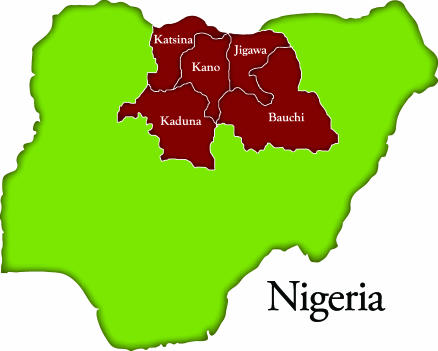
The Five States in Northern Nigeria That Accounted for 51% of All Polio Cases Worldwide in 2006 (Illustration: Anthony Flores; data derived from http://www.polioeradication.org/content/factsheets/Nigeria_12Oct06.pdf).

The report stated further that: “the same virus travelled in 2005 to Yemen, Saudi Arabia, and Indonesia (probably by Muslim pilgrims returning from the Hajj or migrant workers)” [38]. As a result, over 1,500 children were paralyzed. These new cases are genetically linked to the polio virus endemic in northern Nigeria (Figure 3). New cases of polio genetically linked to the wild polio strain from Nigeria have also been recorded in countries as far as the Sudan and Botswana, which were also previously free of polio [38].

## How Can the Health Community Prevent Further Boycotts?

The vaccine boycott in Nigeria was influenced by a complex interplay of factors. These factors included lack of trust in modern medicine, political and religious motives, a history of perceived betrayal by the federal government, the medical establishment, and big business, and a conceivably genuine—albeit misplaced and ineffective—attempt by the local leadership to protect its people.

A recent editorial in *The Lancet* argued that “few data exist on the best way to stop the spread of false information” [39]. One lesson from the Kano boycott is that research is needed to investigate why people have concerns and fears about vaccination, and what steps should be taken to avoid boycotts in the future. Other lessons are discussed below.

### Governments should be sensitive to local politics, especially as they affect health-care delivery

Immunization campaign programs should be a participatory event involving state and local governments, community leaders, and parents. There are three types of community leaders in northern Nigeria—traditional rulers, political leaders, and religious leaders. Traditional rulers acquire their status through succession and their authority is rooted in traditions and customs [40–42]. Political leaders acquire their status through the political process and religious leaders do so on a religious basis. Among the three, the traditional ruler is best placed to represent the interests of children. Community leaders may contribute to the success or failure of health research and delivery [43,44].

### Public awareness campaigns about vaccination are crucial.

These should stress the value of immunization and involve the media. Reaching the community requires radio, television, and folk media (such as local music, theatre, and festivals). Immunization messages can be packaged into songs by local musicians and can be communicated through drama in the language that local people understand.

### Research ethics committees should be established in each local government.

These committees would examine and approve or reject health research in its sphere of influence. Members of these community-based ethics committees should include volunteers who are ready to undergo basic ethics training relevant to their duties. The committees should be under the supervision of, and funded by, the local government's councils, and the committees should work with local medical associations. They should choose their own chairperson and determine their own agenda in line with the national ethics code. Barriers to the formation of local ethics committees include inadequate capacity, funding, and communication.

## Supporting Information

Alternative Language Text S1 Translation of the article into French by E. I. Amenyah (94 KB DOC).

## Abbreviations

ANPP: All Nigeria People's Party
GPEI: Global Polio Eradication Initiative
PDP: People's Democratic Party
SCSN: Supreme Council for Sharia in Nigeria
WHO: World Health Organization
