# Supplementary material for: What Led to the Nigerian Boycott of the Polio Vaccination Campaign?
Source: PLoS Med. 2007 Mar 20;4(3):e73. doi: 10.1371/journal.pmed.0040073 (PMC1831725; doi:10.1371/journal.pmed.0040073)
Supplement: Alternative Language Text S1 — (94 KB DOC). [file pmed.0040073.sd001.doc]

[French translation by Efua Irene Amenyah of the Center for West African

Bioethics Training Program, College of Medicine, University of Ibadan]

**FORUM POLITIQUE**

**Qu’est-ce qui a entrainé le Boycott Nigérian de la Campagne de Vaccination contre la Poliomyélite**

**Ayodele Samuel Jegede**

Ayodele Samuel Jegede est Maître de Conférence au Département de Sociologie, Université d’Ibadan et membre du corps professoral du Programme de Formation en Bioéthique en Afrique de l’Ouest, Faculté de Médecine, Université d’Ibadan, Etat d’Oyo, Nigeria. E-mail : [sayjegede@yahoo.com](mailto:sayjegede@yahoo.com)

**Financement**

Cet article a été écrit lorsque l’auteur était étudiant boursier de Forgaty au Centre Conjoint pour la Bioéthique (JCB) de l’Université de Toronto lors de la session académique 2005/2006.

**Poursuite d’Intérêt**

Le corps enseignant du Centre Conjoint pour la Bioéthique de l’Université de Toronto a révisé la version initiale de cet article. L’auteur affirme qu’il n’existe aucun intérêt de concurrence.

Copyright : © 2007 Jegede. Ceci est un article de libre accès distribué sous les termes de la licence Attribution de création commune qui autorise une utilisation non restrictive, distribution et reproduction par tout moyen, pourvu que l’auteur et la source d’origine soient cités.

La vaccination est un outil essentiel pour prévenir et contrôler la maladie mais son utilisation a suscité des polémiques dans le monde entier [1-6]. Dans cet article, une polémique a été observée autour du programme de vaccination contre la poliomyélite au Nigeria, au sein duquel trois états du Nord ont boycotté en 2003 la campagne de vaccination contre la poliomyélite. Les problèmes causés par le boycott ont été discutés, les implications et les solutions futures. Enfin, il y a eu des recommandations pour prévenir la survenue de telle situation dans l’avenir.

**Méthodes**

Dans le cadre de cet article, nous avons consulté des livres pertinents, des revues (électroniques et en imprimés), des matériels par Internet et des articles de journaux. En particulier, nous avons aussi recherché et utilisé la documentation concernant l’histoire de la vaccination dans le Nord du Nigeria, facteurs responsables du boycott et les questions éthiques découlant du boycott.

**La Campagne Bouter la Polio hors de l’Afrique**

En raison des difficultés rencontrées par certains gouvernements nationaux à faire face aux déclenchements des cas de polio, l’Organisation Mondiale de la Santé (OMS) a lancé en 1988 une Vaste Initiative d’Eradication de la Poliomyélite (GPEI) qui visait à éradiquer totalement la maladie d’ici l’an 2000 ([http://www.polioeradication.org](http://www.polioeradication.org/), [7]). En 1989, l’Assemblée Mondiale de la Santé a approuvé le vaste plan d’action d’éradication de la polio et le Comité Régional de l’OMS pour l’Afrique a adopté la résolution [8].

En 1996, la Région Afrique de l’OMS a intensifié ses stratégies d’éradication de la polio. La même année, le Président Nelson Mandela a lancé la Campagne ***‘‘Bouter la Polio hors de l’Afrique’’*** [9], qui visait à vacciner 50 millions d’enfants cette seule année. Les campagnes de vaccination de masse ont été amplifiées par les Journées Nationales de Vaccination (JNV), la surveillance de graves paralysies flasques, la formation des personnels de santé communautaire au niveau local et les campagnes par la stratégie de porte-à-porte [10 - 11].

La campagne ***‘‘Bouter la Polio hors de l’Afrique’’*** assurait le fonctionnement du système de la chaîne de froid et la formation continue des communautés sur l’importance de la vaccination de routine [9]. A partir de 1997, à travers une alliance avec la Confédération Africaine de Football (CAF), les meilleurs joueurs africains de football participèrent aux campagnes publiques de prise de conscience en distribuant de posters, conduisant des interviews par radio et tenant des sessions publiques d’autographes [8].

A mi-octobre 2003, le GPEI a lancé ce qui était espéré être la dernière attaque contre la Polio, avec un plan pour immuniser plus de 15 millions d’enfants en Afrique de l’Ouest et du Centre. Le GPEI avait des ennuis particuliers concernant la forte prévalence de polio au Nigeria, qui représentait mondialement 45 % de cas de polio et pour la Région Afrique 80 % de cas rapportés en 2003 [12]. Cette forte prévalence était attribuée à une pauvre couverture vaccinale révélée par contrôle durant la précédente campagne. Mais les espoirs de GPEI ont été anéantis à cause du boycott de la campagne de vaccination de polio par les trois états du Nord, à la suite des rumeurs et de la méfiance publique.

**Le boycott dans le Nord du Nigeria**

La confiance publique est indispensable pour promouvoir la santé publique [13]. Une telle confiance joue un rôle important au sein du public en conformité avec les interventions de santé publique, spécialement subséquent aux programmes de vaccination qui ciblent principalement les personnes en bonne santé. Lorsque la confiance publique est sapée, les rumeurs peuvent se répandre et conduire à la méfiance des interventions de santé.

Au Nord du Nigeria en 2003, les responsables politiques et religieux des Etats de Kano, Zamfara et Kaduna ont dû interrompre la campagne de vaccination en appelant les parents de ne pas permettre que leurs enfants soient vaccinés. Ces responsables argumentent que le vaccin pourrait être contaminé par des agents non-fertilisants (hormone estradiol), cancérigènes et le VIH.

Dans un article rapporté par News24.com, un site de Nouvelles en ligne sud africain, Sule Ya’u Sule, s’adressant au Gouverneur de Kano, est cité ainsi : *‘‘Depuis le 11 septembre 2001, le monde musulman est devenu soupçonneux de tout mouvement venant du monde occidental…. Nos populations sont réellement inquiètes du vaccin de polio’’* [13]. Dans le même article, Datti Ahmed, un médecin basé à Kano qui est à la tête d’un groupe musulman très influent, la Cour Suprême de la Shari’a du Nigeria (SCSN), est cité en disant que les vaccins de polio étaient ‘‘corrompus et souillés par les mauvais esprits de l’Amérique et de leurs alliés occidentaux’’. Ahmed continua en disant : *‘‘Nous croyons que les Hitler des jours actuels ont délibérément frelaté les vaccins oraux de la polio avec les médicaments non-fertilisants et des virus qui sont réputés être la cause du SIDA’’* [13].

Le *New York Sun* a rapporté que cette peur des vaccins de la polio au Nord du Nigeria *‘‘devient populaire à cause de la guerre en Iraq’’* [14]. Ali Guda Takai, un médecin de l’OMS qui a enquêté sur les cas de polio dans l’état de Kano, raconta dans le *Baltimore Sun*, *‘‘Ce qui se passe au Moyen Orient a aggravé la situation. Si l’Amérique combat les peuples du Moyen-Orient, la conclusion est qu’elle combat les Musulmans’’* [15].

Embarrassé par la tournure politique du boycott, un éminent érudit musulman, Sheikh Yusuf Al-Qaradawi, Président du Conseil International Fiqh, disait : ‘‘*En fait, j’étais complètement étonné à propos de l’attitude de nos confrères lettrés de l’état de Kano à propos du vaccin de polio. Je désapprouve leur opinion, concernant la validité de tel vaccin, le point de vue de l’Islam est clair comme la lumière du soleil’’* [16]. Sheikh Qaradawi disait que le même vaccin de polio a été efficace dans plus de 50 pays musulmans et blâmait le SCSN de créer une image négative de l’Islam : *‘‘ils déforment l’image de l’Islam et le font apparaître comme s’il est contradictoire à la science et au progrès médical’’* [16].

**Contexte du Boycott**

**Contexte historique**

Le boycott de la vaccination contre la polio ne devrait pas être considéré comme un cas isolé mais plutôt dans son contexte historique des services des soins de santé classiques dans le Nord du Nigeria. Généralement, le taux d’utilisation des services des soins de santé classiques dans la région a toujours été faible. En exemple, la comparaison des taux d’utilisation au Sud contre le Nord du Nigeria était de 50 % contre 18 % en 1990 (i.e. la moitié de la population du Sud utilise les services des soins de santé classiques comparée à moins d’un cinquième dans le Nord), 60 % contre 11 % en 1999 et 64 % contre 8 % en 2003 [17 - 19].

Autres facteurs historiques qui alimentent le boycott de la vaccination contre la Polio comportent la contrôle de la population et de la fertilité. Dans les années 1980, l’administration du Président Babangida adoptait une politique de population qui fixa la limite de quatre (4) enfants par femme. Des populations reliaient cette campagne de régulation de la population à la vaccination, croyant que la vaccination constituait le moyen par lequel le gouvernement pouvait réduire la population [20]. Cette croyance n’était seulement pas restreinte au Nord du Nigeria - des opinions similaires étaient également exprimées dans certaines communautés dans le Sud du Nigeria.

En exemple, dans une étude anthropologique conduite au Nigeria [21], un participant adulte affirmait : *‘‘les personnes soutiennent la rumeur que la vaccination est un moyen secret de contrôler la population’*’. Une jeune participante disait: *‘‘des personnes disent que la vaccination fait partie des méthodes utilisées pour contrôler le nombre d’enfants qu’une femme peut avoir’’*.

Un autre important facteur qui jouait un rôle dans le boycott du vaccin de polio était la méfiance générale à l’endroit des agressifs programmes de vaccination de masse dans un pays où l’accès aux soins de santé primaire n’était pas aisément disponible [15]. Dans son rapport dans le *Baltimore Sun*, John Murphy écrivait : *‘‘c’est la vaccination de masse agressive de porte-à-porte, qui a lacéré les infections de polio dans le monde entier souleva ainsi des suspicions. Dans la perspective nigériane, offrir des médicaments gratuits est souvent aussi inhabituel qu’un étranger fasse du porte-à-porte en Amérique pour donner des billets de 100 US$. Cela n’a pas de sens dans un pays où la population lutte au niveau des centres de santé locaux pour obtenir les médicaments et les traitements les plus élémentaires* [15]’’.

**Contexte politique**

Au Nigeria, les états ont le contrôle administratif de la politique de santé pour les soins de santé aux niveaux primaire et secondaire alors que le gouvernement fédéral s’occupe des soins de santé au niveau tertiaire. Bien que le gouvernement fédéral ait élaboré une politique de santé pour toute la nation, la vaccination est sous la responsabilité du système des soins de santé primaire dans chaque état. C’était pourquoi le gouvernement de l’état de Kano était en droit d’émettre une directive pour interrompre l’opération de vaccination planifié par le gouvernement fédéral.

Le Nigeria étant une société multipartite, les parties d’opposition exercent leurs droits politiques en défiant constamment le parti au pouvoir. Après les élections présidentielles de Mai 2003, le Parti de Tous les Peuples du Nigeria (ANPP) conduit par le Général Muhamadu Buhari, dictateur militaire et une fois Chef d’Etat instruira un cas à la Cour Suprême du Nigeria défiant la victoire du Président Olusegun Obasanjo du Parti Démocratique des Peuples (PDP) [16]. Aussi, l’état de Kano étant sous le contrôle de l’ANPP défiait l’opération de vaccination contre la polio organisée par le PDP contrôlant le gouvernement fédéral.

Le Nigeria traverse une transition politique à partir d’un régime militaire dirigé par un Nordiste à une démocratie conduite par un Sudiste. Jusqu’en 1999, le Nord a gouverné le pays pendant plus de 30 ans sur les 46 ans d’indépendance. Depuis le début du nouveau système démocratique adopté par le gouvernement en 1999, le pouvoir est passé au Sud (précisément le sud ouest). Ces changements ont créé des tensions politiques entre le Sud et le Nord. Ces tensions pourraient justifier pourquoi les responsables religieux des états du Nord, qui ont boycotté la campagne de vaccination contre la polio, croyaient que le gouvernement fédéral dirigé par un Sudiste agissait dans l’intérêt des pouvoirs occidentaux. Les parties Nord et Sud du pays ont eu différentes expériences coloniales. Pendant que le Nord était colonisé par les djihads islamiques, le Sud était colonisé par les Anglais. Ces expériences coloniales sont responsables des différences politiques entre le Nord et le Sud et des différentes attitudes face à la médecine moderne.

**Essai de Trovan**

Les soupçons à propos des interventions occidentales de santé étaient déjà en circulation dans le Nord du Nigeria, bien avant le boycott de la vaccination contre la Polio, à la suite de l’***‘‘Essai de Trovan’’*** de Pfizer en 1996 [22 - 24]. L’essai était discuté en détail dans un *BMJ* intitulé *‘‘Pfizer accusé de tester un nouveau médicament sans approbation éthique’’* [22].

En bref, le *BMJ* rapporte qu’en 1996 Pfizer a envoyé une équipe à Kano durant l’épidémie de Méningite méningocoque. Afin de tester l’efficacité de son nouvel antibiotique trovafloxacin (Trovan), l’équipe conduisit un essai sans label sur 200 enfants - la moitié recevait le traitement normal standardisé de méningite, ceftriaxone et l’autre moitié recevait trovafloxacin. Cinq des enfants ayant reçu trovafloxacin moururent, de même que six qui ont reçu ceftriaxone. Le *BMJ* rapportait : *‘‘The Washington Post avait examiné l’essai et prétendait qu’au moins un enfant était extrait du médicament expérimental et il lui est administré le médicament standardisé lorsqu’il est clair que sa condition ne s’améliorait pas - ce qui était contraire aux directives éthiques’’*. Le *BMJ* rapportait que le Ministre Nigérian de la Santé a désigné un panel fédéral d’enquête pour déterminer si l’essai était conduit légalement et si c’était le cas, s’il est moralement conforme.

Le 7 mai 2006, *The Washington Post* rapportait qu’il a été privilégié de consulter un rapport secret d’enquête du panel, qui prétendait que Pfizer a entrepris un *‘‘essai illégal d’un médicament non enregistré’’* et la compagnie a recruté des enfants pour l’essai de Trovan [25]. En réponse à la fuite du rapport, Pfizer émit une déclaration de presse en disant : *‘‘Pfizer est confiant que son personnel n’ait pas associé à l’étude clinique de Trovan - conduite à Kano, Nigeria durant l’épidémie de méningite de 1996 - ne mettra jamais la santé du patient à risque car la compagnie a agi dans les meilleurs intérêts des enfants impliqués dans l’étude, en utilisant la meilleure connaissance médicale disponible’’* [26].

En 2001, 30 familles nigérianes ont poursuivi Pfizer en justice devant une cour fédérale à New York [27]. La requête prétendait : *‘‘Pfizer opta de sélectionner des enfants pour participer à une expérimentation médicale d’un nouveau médicament, non testé et non approuvé sans obtenir en premier leur consentement éclairé’’*. Au cours des 4 années qui ont suivi, Pfizer a argumenté que le cas ne devrait pas du tout être entendu dans une cour des Etats Unis [28]. En août 2005, le Juge William H. Pauley III du district Sud de New York approuvait en déclarant que le Nigeria et non les Etats Unies, était la place appropriée pour tenter une poursuite judiciaire contre la conduite de l’essai de Trovan de Pfizer (Abdullahi v. Pfizer Inc., S.D.N.Y., N°. 01 CIV 8118, 8/9/05) [29].

Selon le rapport de John Murphy dans le *Baltimore Sun*, l’essai Trovan pouvait avoir laissé des Nigérians méfiants des interventions occidentales : *‘‘certaines des peurs avec Kano concernent le contenu du vaccin par rapport à son expérience avec le géant pharmaceutique Pfizer Inc.’’* [15].

**La réponse fédérale au Boycott de la Polio**

En réponse au tollé général concernant le vaccin de la polio, le gouvernement fédéral du Nigeria met en place le 29 octobre 2003 un comité technique pour évaluer la sûreté du vaccin de la polio en envoyant des exemples du vaccin dans des laboratoires étrangers pour des tests. Le rapport du comité était, cependant, rejeté par la Cour Suprême de la Shari’a du Nigeria, qui prétendait que la communauté musulmane n’était pas correctement représenté dans le comité.

En réponse à cette allégation, le gouvernement fédéral désigna un autre comité technique, qui comprenant des membres choisis de la Jama’attu Nasril Islam, pour confirmer de nouveau plus tard la sûreté du vaccin. Malgré cela, la Cour Suprême de la Shari’a rejeté encore les membres, en demandant que ce soit inclus leurs propres membres. Justifiant leur continuelle opposition au vaccin de la polio malgré l’alarme de prévalence élevée de 30 % de cas de polio. En janvier 2004, le gouvernement de l’état de Kano argumentait : *‘‘…le moindre des deux maux est de sacrifier 2, 3, 4, 5 même 10 enfants par la polio que de permettre que des centaines de milliers ou probablement des millions d’enfants filles ne soient éventuellement rendus stériles’’* [30].

Bien que la vérité sur la rumeur que le vaccin de la polio contenait le VIH et les agents non-fertilisants et cancérigènes n’ait jamais été élucidée, le manque de confiance parmi l’ensemble de la population au Nord du Nigeria concernant l’efficacité des médicaments occidentaux demeurait. Tous les efforts entrepris par le gouvernement fédéral pour dissiper les rumeurs ont été rejetés. En exemple, l’éminent responsable islamique, Datti Ahmed, argumentait en avril 2004 que : *‘‘la SCSN [Cour Suprême de la Shari’a du Nigeria] nourrissaient de solides doutes sur la sûreté de nos populations, non moins, parce que, de notre récente expérience dans le scandale de Pfizer, nos populations étaient utilisés comme des cobayes avec l’approbation du Ministère Fédéral de la Santé et les agences correspondantes des Nations Unies’’* [30].

Cette situation était finalement résolue en juillet 2004 par le dialogue avec les responsables religieux qui ont joué un rôle significatif dans le processus. Le gouvernement fédéral avait invité les responsables politiques et religieux à de séries de réunions afin de trouver une solution à la situation. L’OMS et l’UNICEF ont également joué un rôle dans la cessation de la crise. Ces réunions ont conduit à un consensus en février 2004 pour accepter la demande de la SCSN de tester indépendamment le vaccin dans un pays musulman. En février 2004, le gouvernement nigérian envoya des représentants de l’Etat et des responsables religieux en Afrique du Sud, en Indonésie et en Inde pour observer le test du vaccin de la polio et de *‘‘ramener des évidences’’* qu’il n’était pas contaminé par le VIH [30 - 31].

En défense des 11 mois de boycott, le gouverneur de l’état de Kano, Ibrahim Shekarau, réaffirmait que leur décision était influencée par l’insatisfaction des résultats du test par les équipes du gouvernement fédéral. Satisfait par la qualité et le processus de la production du vaccin de polio, l’équipe de l’état de Kano retournait avec un sceau d’approbation de Biopharm, une compagnie indonésienne qui sera recommandée pour être plus tard le nouveau fournisseur des vaccins de polio principalement pour les états musulmans et peut-être le reste du pays [32]. L’Indonésie est un pays musulman en qui les responsables musulmans du Nigeria crurent pour avoir testé le vaccin de polio.

Deux mois après, l’état de Kano reprit son programme de vaccination, près de 150 musulmans religieux et chefs traditionnels du Tchad, Cameroun, Niger, Togo, Bénin et Burkina Faso se rencontraient à Kano le 22 septembre 2004 pour discuter des solutions futures pour ce qui est de la campagne de vaccination contre la polio. La réunion était accueillie par l’OMS et l’UNICEF et son but était d’informer les responsables religieux et traditionnels sur les problèmes qui affectent les enfants, avec un accent sur la polio. La réunion a également partagé les connaissances et les expériences et produisit un calendrier de plaidoyer pour garantir que de corrects messages soient fournis aux populations [33].

**Le communautarisme africain**

Le gouvernement fédéral, ayant perdu sa confiance publique dans un état - une perte de confiance suscitée par les responsables musulmans – a dû handicaper la prestation des services des soins de santé. Dans plusieurs contrées en Afrique, la communication et l’autorité échappent aux responsables des communautés qui sont les gardiens et les décideurs. Dans la communauté Haussa au Nord du Nigeria, les dirigeants traditionnels ont les pouvoirs résultant à la fois de la culture et de la religion. Ceci leur donne l’occasion d’assumer des rôles politiques et religieux. Ils règnent à travers les conseils traditionnels et les conseils islamiques, sous la supervision du gouvernement local, en ayant leur personnel du bureau recruté du gouvernement de l’état.

Le système politique moderne dans le Nord du Nigeria repose sur cette structure pour atteindre et mobiliser la population. Ceci est important dans le sens qu’il montre un type de communautaris-me où l’autonomie individuelle ne peut être séparée de celle de la communauté. Les responsables de l’état de Kano croyaient qu’ils agissaient pour protéger l’intérêt de leur population, mais malheureusement, ils ont fini par causer des dommages à la cohésion nationale.

**Les efforts futurs de vaccination**

Une nouvelle éruption de cas de polio a été rapportée dans l’état de Kano en octobre 2003. La BBC rapportait qu’à cause de cette nouvelle éruption, une nouvelle variété du virus de polio était découverte dans d’autres parties du pays [35]. Même plusieurs années après le boycott, les éruptions de cas de polio demeurent une régulière occurrence au Nigeria, et ceux-ci montrent des formes de résistance aux vaccins. Dans plusieurs autres endroits du monde, lorsque 3 ou 4 doses de vaccin de polio administrées à un jeune enfant est suffisant pour lui offrir la protection, au Nigeria, avec plusieurs types de virus de polio circulant, un enfant de moins de cinq ans a besoin de se faire vacciner près de 8 fois et même plus [23].

Le cas récent d’un garçon de 18 mois à Nasarawa dans l’état de Kano montre qu’il y a un soupçon continu à propos du vaccin de polio au Nigeria. Le garçon était probablement supposé avoir la polio car il a perdu ses jambes après avoir été diagnostiqué avec de graves paralysies flasques. Sa mère confirmait qu’il n’avait jamais été vacciné parce que son père n’avait pas permis [23]. Il y a encore à réaliser une grande entreprise pour avoir la compréhension de la population que le vaccin est sain [36].

En septembre 2006, le rapport du Comité de l’OMS pour l’Afrique indiquait que *‘‘il continue d’avoir la propagation de dangereux virus de polio de très haute sensibilité dans le reste des zones endémiques des pays dans la région. Le nombre des cas de polio confirmés dans le Nord du Nigeria a plus que doublé dans les 5 premiers mois de 2006 comparé à la même période de 2005. Le Nigeria justifie maintenant de plus de 80 % du fardeau global de polio de 2006’’* [37 - 38].

De nouveaux cas de polio ont été récemment rapporté dans beaucoup de pays de l’Afrique de l’Ouest et du Centre qui étaient précédemment déclarés sans cas de Polio : Bénin, Burkina Faso, Cameroun, République Centrafricaine, Tchad, Côte d’Ivoire, Ghana et Togo. Le *Lancet* rapporta récemment que *‘‘le stéréotype 1 du virus de polio a causé de très sérieuses éruption à larges échelles durant 2004 en Afrique de l’Ouest et du Centre (se répandant à partir du Nigeria) où la vaccination a été refusée pour des raisons politiques et religieuses, une peur de contamination délibérée du vaccin avec le VIH et les agents infertiles’’* [38].

Le rapport affirmait plus tard que : *‘‘le même virus a été trouvé en 2005 au Yémen, en Arabie Saoudite et en Indonésie (probablement par les pèlerins musulmans revenant du Hadj ou des travailleurs migrants)’’* [38]. Comme résultat, plus de 1500 enfants étaient paralysés. Ces nouveaux cas sont génétiquement liés au virus de polio endémique dans le Nord du Nigeria. De nouveaux cas de polio, génétiquement liés au virus de polio sauvage franchissant le Nigeria, ont été également enregistrés dans de lointains pays comme le Soudan et le Botswana qui ont été précédemment déclarés sans cas de Polio également [38].

**Comment la communauté de santé peut-elle prévenir les futurs boycotts**

Le boycott du vaccin au Nigeria a été influencé par complexes facteurs interactifs. Ces facteurs comprenaient le manque de confiance dans la médecine moderne, les raisons politiques et religieuses ; une histoire que certains considèrent comme une trahison du gouvernement fédéral, des établissements des soins de santé et, en théorie, une grande et authentique affaire - bien que mal placé et inefficace - ont été gérés par les responsables locaux pour protéger leur population.

Une récente éditoriale dans le *Lancet* argumentait que : *‘‘peu de données existe sur le meilleur moyen d’arrêter la propagation de fausse information’’* [39]. Une leçon du boycott de Kano est qu’il y a nécessité d’une recherche pour enquêter pourquoi les populations ont des inquiétudes et des peurs au sujet de la vaccination et quelles mesures devraient-elles être entreprises pour éviter des boycotts dans le futur. D’autres leçons sont discutées ci-dessous.

**Gouvernements doivent être attentifs aux politiques locales, particulièrement celles qui affectent la prestation des soins de santé**

Les campagnes des programmes de vaccination devraient être un événement participatif impliquant l’état, les gouvernements locaux, les responsables communautaires et les parents. Il existe trois types de responsables communautaires dans le Nord du Nigeria - chefs traditionnels, responsables politiques et responsables religieux. Les chefs traditionnels acquièrent leur statut par la succession et leur autorité est enracinée dans les traditions et les coutumes [40 - 42]. Les responsables religieux acquièrent leur statut sur une base religieuse et les responsables politiques agissent pareillement par le truchement du processus politique. Parmi les 3, le chef traditionnel est le mieux placé pour représenter les intérêts des enfants. Les responsables communautaires peuvent contribuer au succès ou à l’échec des recherches et des prestations de santé [43].

**Campagnes de prise de conscience publique concernant la vaccination sont cruciales**

Celles-ci devraient insister sur le bénéfice de la vaccination et le média devrait être impliqué. Pour atteindre la communauté, il faut la radio, la télévision, le média folklorique (telle que la musique locale, le théâtre et les festivals). Les messages de vaccination peuvent être ensachés dans les chansons locales par les musiciens locaux et peuvent être communiqués au moyen du théâtre dans la langue locale que la population locale comprend.

**Comités éthiques de recherche devraient être installés au niveau de chaque gouvernement local**

Ces comités pourront examiner et approuver ou rejeter les recherches de santé dans leur cercle d’influence. Les membres des comités communautaires éthiques devront inclure des volontaires, prêts à poursuivre des formations initiales en éthique, appropriées pour leurs responsabilités et devront travailler avec l’association médicale locale. Ces comités devront être sous la supervision de, et financés par, les conseils du gouvernement local. Ils devront choisir leur propre président et déterminer leur propre agenda en conformité avec le code national éthique. Les barrières à la mise en place des comités éthiques locaux sont la capacité, le financement et la communication inadaptés.

**Remerciement**

Nous remercions les Professeurs Melissa Leach et Mariam Yahaya de l’Institut de Développement des Etudes (IDS), Université de Sussex ; pour avoir procuré les informations utilisées dans cet article.

**Références**

1. Lurie P., Bishaw M., Chesney M.A., Cooke M., Fernandes M.E., Hearst N. et al., 1994 ; *Ethical, behavioural and social aspects of HIV vaccine trials in developing countries*. *JAMA* ; 271 : 295 - 301.
2. Temoshok L.R., 1994 ; *Behavioral research contributions to planning and conducting HIV vaccine efficacy studies*. *AIDS Res. Hum Retroviruses* ; 10 (Suppl 2) : S 277 - S196 [ISI][Medline].
3. Berkley S., 1998 ; *HIV vaccine development for the world: an idea whose time has come?* *AIDS Res. Hum Retroviruses* 14 (Suppl 3) : S191 - S196 [ISI][Medline].
4. Excler J.L., Beyer C., 2000 ; *Human immunodeficiency virus vaccine development in developing countries: are efficacy trials feasible*. *J. Hum Virol*, 3 : 193 - 214 [ISI][Medline].
5. Mbidde E., 1998 ; *Bioethics and local circumstances*. *Sciences*, 279 : 155.
6. Mugerwa R.D., Kaleebu P., Mugyeny P., Katongole-Mbidde E., Byaruhange R., Sa,ata R.A. et Ellner J.J., 2002 ; *First trial of the HIV-1 vaccine in Africa : Uganda experience*. *BMJ* : 324 : 226 - 229.
7. Assemblée Mondiale de la Santé, 1988 ; *Eradication complète de la Poliomyélite d’ici l’an 2000* : Résolution de la 41^e^ Assemblée Mondiale de la Santé. Genève, Suisse ; Organisation Mondiale de la Santé (Résolution WHA 41.28).
8. Letore D., *Campaign to kick polio out of Africa*. *Africa Recovery*, Décembre 1998. 12 (3) : 18 - 19. http://www;anc.org.za/ancdocs/history/mandela/1996/-sp0802.html
9. Comité Régional pour l’Afrique. *Le Programme de Vaccination Elargi : objectifs de contrôler la maladie, le compte à rebours a commencé* --- Résolutions du 45^e^ Comité Régional pour l’Afrique. Brazzaville, Congo : Organisation Mondiale de la Santé, 1995 (Résolution AFR/RC45/R5).
10. Organisation de l’Unité Africaine. *Déclaration de Yaoundé sur l’éradication de la Poliomyélite en Afrique*. In : Mesures de la 32^e^ Session Ordinaire de la Réunion de l’Organisation de l’Unité Africaine. Yaoundé, Cameroun. Organisation de l’Unité Africaine 1996 (AHG/Déclaration 1 [XXXII].
11. CDC, 30 avril 2004 ; *Progress toward poliomyelitis* *eradication* - Nigeria, Janvier 2003 - Mars 2004, *Morbidity Mortality Weekly Report*, 53 (16) : 343 -346.
12. Renne E., 8 Juin 2006 ; *Perspectives on polio and immunization in northern Nigeria*. *Sciences sociales et* *Médecine*, [Epub. Ahead of print]. PMId : 16765498 [PubMed - comme fournisseur de l’éditeur].
13. Anonyme, 11 février 2004 ; *Vaccine boycott spread Polio*. *News 24.com South* *Africa* -. <http://www.news24.com/News24/Africa/Features/0,,2-11-37_1481952,00.html>
14. <http://www.danielpipes.org/article/2644>
15. Murphy J., *Distrust of US foils effort to stop crippling disease*. <http://www.baltimoresun.com/news/nationworld/balpolio0104,1,6396183.story?ctrack=1&cset=true>
16. Odutola A., 20 janvier 2004 ; *Nigeria Polio, politics and power play*. Afro-Nets. <http://www.afronets.org/archive/2004/msg00069.php>
17. Mesures, *Etude Démographique et de Santé*, USAID, 1990.
18. Mesures, *Etude Démographique et de Santé*, USAID, 1999.
19. Mesures, *Etude Démographique et de Santé*, USAID, 2003.
20. Odumosu O., Robert F.N., Jegede A.S., 1996 : *‘‘Regional differential and implementation of the National Policy on population : Pilot study of Borno and Oyo states of Nigeria’’*. In Phillips AO et Ajakaiye DO (eds). Population-Environment Interactions in Nigeria: NISER *Studies in Sustainable Development in Nigeria*, NISER, Ibadan. 7 - 42.
21. [http://www.ids.ac.uk/IDS/KNOTS/PDFs/VaccJegedeNigeria.pdf#search=%22Regional%20differential%20and%20implementation%20of%20the%20National%20Policy%20on%20population%3A%20Pilot%20study%20of%20Borno%20and%20Oyo%20states%20of%20Nigeria%22](http://www.ids.ac.uk/IDS/KNOTS/PDFs/VaccJegedeNigeria.pdf" \l "search="Regional differential and implementation of the National Policy on population%3A Pilot study of Borno and Oyo states of Nigeria")
22. Wise J., 2001 ; *Pfizer accused of testing new drug without ethical approval*. *BMJ,* 322: 194.
23. Bomford A., *Nigeria’s Struggle to Beat Polio*. *BBC News*. <http://news.bbc.co.uk/2/hi/africa Downloaded 25/4/2006>.
24. Stephens J., *As Drug Testing Spreads, Profits and Lives Hang in Balance*. *Washington Post*, 17 décembre 2000 ; Page A01.
25. Lenzer J., 2006 ; *Secret report surfaces showing that Pfizer was at fault in Nigeria Drug tests*. *BMJ,* 332 : 1233.
26. <http://mediaroom.pfizer.com/intex.php?s=press_releases&item=55>
27. Kovac C., 2001 ; *Nigeria to sue US drug company over meningitis treatment*. *BMJ* 323 : 592.
28. Angell M., *The Body Hunters*. <http://www.nybooks.com/articles/18301>
29. Pfizer Wins Bid to Dismiss Nigerians’ Suit Over Injuries Related to Trials of Antibiotic. <http://subscript.bna.com/SAMPLES/pld.nsf/85256269004a9921852562540065050d/71087d5c0113cc678525706e00824260?OpenDocument>
30. Yahaya M., Polio Vaccines – difficult to swallow: The story of a controversy in Northern Nigeria *IDS Working Paper* 261. <http://www.ids.ac.uk/IDS/KNOTS/PDFs/VaccYahyaNigeria.pdf>
31. <http://www.theglobalfund.org/programs/news_summary.aspx?newsid=46countryid=IND&lang>=
32. <http://www.boston.com/news/world/articles/2004/07/20/ban_on_polio_vaccine_lifted_in_nigeria_state/>
33. Cameron Duodu, *The fear of vaccines: fear that a vaccine used by the WHO to combat Polio can cause sterility in both men and women, has spurred some Northern Nigerian states to block a crucial vaccination campaign that is aimed at eradicating the disease from West Africa*. *Nouvelle Africaine* - Avril 2004.
34. Tangwa G.B., (1996) ; *Bioethics: An African Perspective*. *Bioethics*, 1996; 10(3): 183 -200.
35. Barnaby P., *Nigeria drug trial fear*. *BBC News/Africa*. <http://news.bbc.co.uk/1.hi.world/Africa/1220033.stm>
36. <http://www.lifenetradio.org/lnnews/inn0915.html>
37. WHO, *Eradication de la Polio dans la région Afrique : Rapport de progrès*. Organisation Mondiale de la Santé, *Comité Régional pour l’Afrique*. AFR/RC56/INF.DOC/1, 7 juillet 2006.
38. Kimman T.G. et Boot H., 2006 ; *The polio eradication effort has been a great success – let’s finish it and replace it with something even better*. *Lancet : Maladies infectieuses*. vol. 6 : 675 - 678.
39. L’Editorial de Lancet. 2006 ; *Who would you believe?* *Lancet* 386 - 340.
40. Casterllano M.B., Janvier 2004 ; *Ethics of Aboriginal Research*.  *Journal de la Santé Aborigène*. 98 - 114.
41. Tangwa G.B., 2000 ; *The Traditional African Perception of a Person: Some Implications for Bioethics*. *Hasting Center Report*, 30(5) : 39 - 43.
42. louw D.J., *Ubuntu: an African assessment of the religious other*. <http://www.bu.edu.wcp/Papers/Afr/AfriLouw.htm Downloaded 28/04/2006>.
43. Zion D., Avril 2005 ; *Community without communitarianism: HIV/AIDS research, prevention and treatment in Australia and the developing world*. Monash Bioeth Rev. 24(2) : 20 - 31.
